# Severe palmoplantar keratoderma: a cutaneous complication from sub-optimally controlled type 2 diabetes

**DOI:** 10.1530/EDM-24-0088

**Published:** 2025-01-09

**Authors:** Fatima Iqbal, Kevin Phan, Wah N Cheung

**Affiliations:** ^1^ Department of Diabetes and Endocrinology, Westmead Hospital, Sydney, New South Wales, Australia; ^2^ College of Medicine, Alfaisal University, Riyadh, Saudi Arabia; ^3^ Department of Dermatology, Westmead Hospital, Sydney, New South Wales, Australia; ^4^ Discipline of Medicine, Randwick Clinical Campus, University of New South Wales (UNSW) Sydney, Sydney, New South Wales, Australia; ^5^ Faculty of Medicine and Health, University of Sydney, Sydney, New South Wales, Australia

**Keywords:** palmoplantar keratoderma, type 2 diabetes

## Abstract

**Summary:**

Palmoplantar keratoderma (PPK), characterised by excessive epidermal thickening of the skin on the palms and/or plantar surfaces of the feet, can be hereditary or acquired. Here, we report a case of a 53-year-old woman with a history of sub-optimally controlled diabetes mellitus presenting with fevers and decreased Glasgow Coma Scale (GCS) to a tertiary hospital. She was diagnosed with diabetic ketoacidosis (DKA), with blood glucose at 40 mmol/L and ketones at 7 mmol/L, in the setting of a methicillin-sensitive *Staphylococcus aureus* necrotising soft tissue back infection. Her medical history included diabetes managed with insulin but no engagement with an endocrinologist or allied health support. Examination revealed an infected, necrotic back wound on her left mid-upper back that required surgical debridement and broad-spectrum IV antibiotics. In addition, she exhibited marked plantar keratoderma and onychogryphosis, reportedly present and worsening over approximately two years. She was prescribed 40% urea cream twice daily, resulting in gradual sloughing of the hyperkeratotic skin within a few weeks. Her HbA1c was 10.4%, and she tested negative for diabetes antibodies, indicating type 2 diabetes. Treatment included an insulin–dextrose infusion until DKA resolved, followed by twice daily insulin degludec/aspart (Ryzodeg 70/30) and metformin. The PPK was attributed likely secondary to sub-optimally managed diabetes.

**Learning points:**

- Diabetes mellitus has multiple complications, including rare dermatologic manifestations such as PPK.
- This case illustrates the importance of thorough skin assessments in patients with diabetes, particularly those that have a history of sub-optimal diabetes control.
- A multidisciplinary approach, integrating dermatology, endocrinology and allied health services such as podiatry, is essential in managing diabetes-related complications, improving patient quality of life and preventing further complex manifestations.

## Background

Palmoplantar keratoderma (PPK) is a heterogeneous group of disorders presenting with excessive thickening of the skin on the palms and/or plantar surfaces of feet (1). It may be hereditary (autosomal dominant or recessive) or acquired, associated with systemic conditions such as uncontrolled diabetes, infections, inflammatory skin conditions or specific medications. Inherited forms can exhibit diffuse, focal or punctate patterns, sometimes as part of syndromes involving multisystemic features. The prevalence of PPK is estimated to be up to 4.4 per 100,000 people (2). Genetic mutations associated with PPK often involve keratins, desmosomes, desmogleins, loricrin, gap junctions and other structural proteins.

## Case presentation

A 53-year-old Caucasian woman presented to a large tertiary hospital with a blood glucose level (BGL) of 40 mmol/L, ketones at 7 mmol/L, fevers and a decreased level of consciousness. She also had an infected wound on the left side of her mid-upper back and severe hyperkeratotic skin on the soles of her feet. She had a history of diabetes, although the type was unclear at the time of presentation, for which she had previously been treated with insulin. Her background included intellectual impairment, with no other significant medical or family history.

She was admitted for the management of diabetic ketoacidosis and septic shock, likely secondary to the infected back wound. She required ICU admission for intubation to protect her airway, along with an insulin–dextrose infusion, inotropic support and broad-spectrum antibiotics to manage her septic shock.

During her hospital stay, she was assessed by the dermatology team for marked hyperkeratosis on the plantar surfaces of both feet, which she reported had been present for at least two years and was gradually worsening. She was diagnosed with acquired PPK, likely due to sub-optimally controlled diabetes.

**Figure 1:**
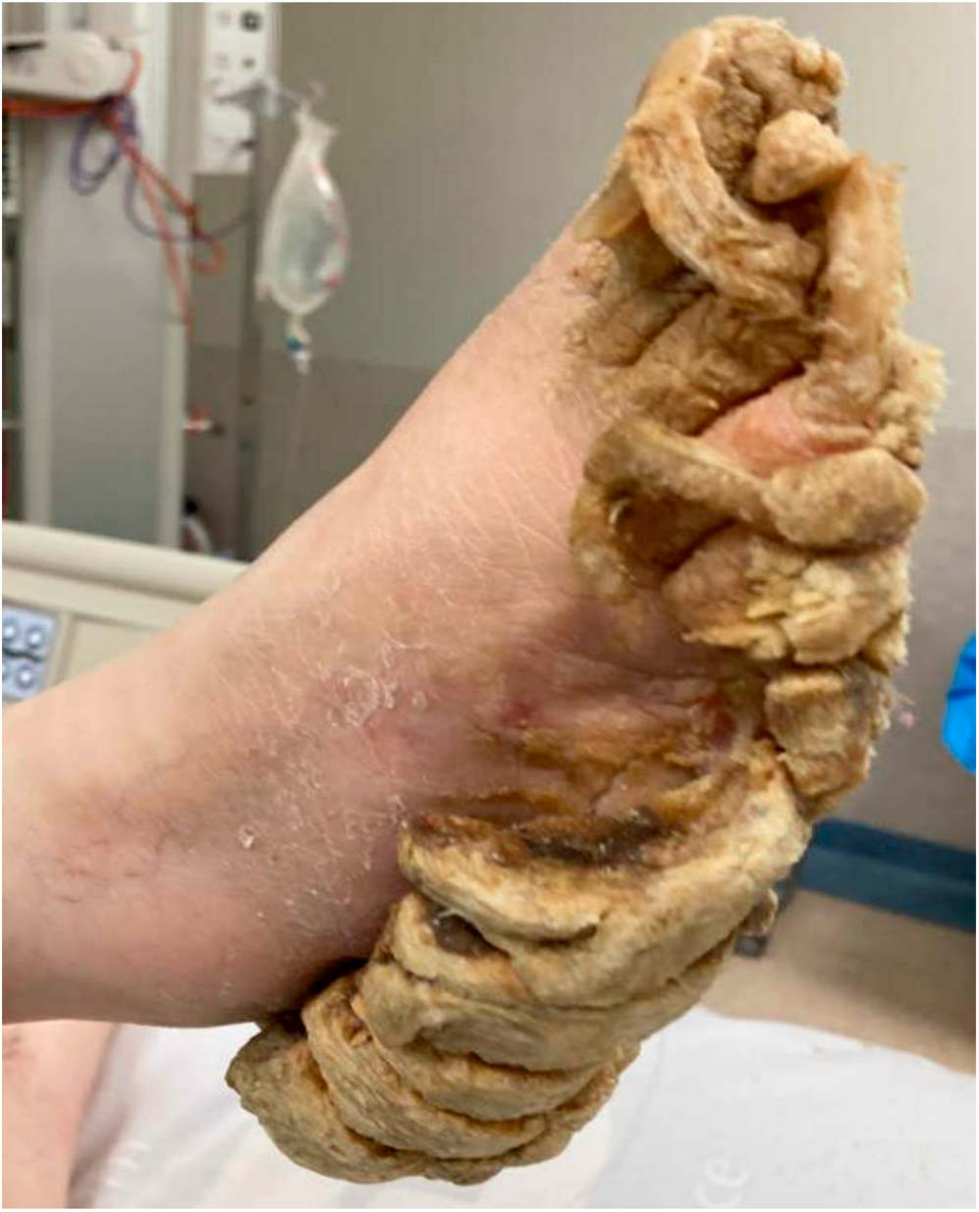
Right foot initial presentation.

On examination, it was noted that pronounced, thick hyperkeratosis and keratoderma with a diffuse pattern covered the entire plantar surfaces of both feet. The hyperkeratosis was so extensive that it nearly encased the lateral and medial aspects of her feet in a pseudo-transgrediens manner (Fig. 1). Concurrent onychogryphosis was noted. Minimal PPK was evident on her hands. She showed no other features suggestive of a syndrome, such as ectodermal abnormalities in hair shafts or teeth, nor any history of cardiomyopathy or malignancies.

## Investigation

She underwent surgical debridement of the back wound, which revealed a methicillin-sensitive *Staphylococcus aureus* necrotising soft tissue infection. This was treated with flucloxacillin.

Her HbA1c was 10.4%, and both anti-insulin and anti-glutamic acid decarboxylase antibodies were negative, indicating a likely diagnosis of type 2 diabetes.

## Treatment

She was weaned off the insulin–dextrose infusion once she was extubated and able to tolerate an oral diet. She was then transitioned to twice daily Ryzodeg, with 12 units in the morning and 10 units in the evening, along with metformin 1,000 mg twice daily.

For her PPK, she was prescribed 40% urea cream, applied twice daily. Acitretin was considered as a systemic option to help reduce the hyperkeratosis; however, due to her critical condition and recent intubation, the risks were deemed to outweigh the benefits, so this systemic retinoid was not prescribed.

## Outcome and follow-up

**Figure 2:**
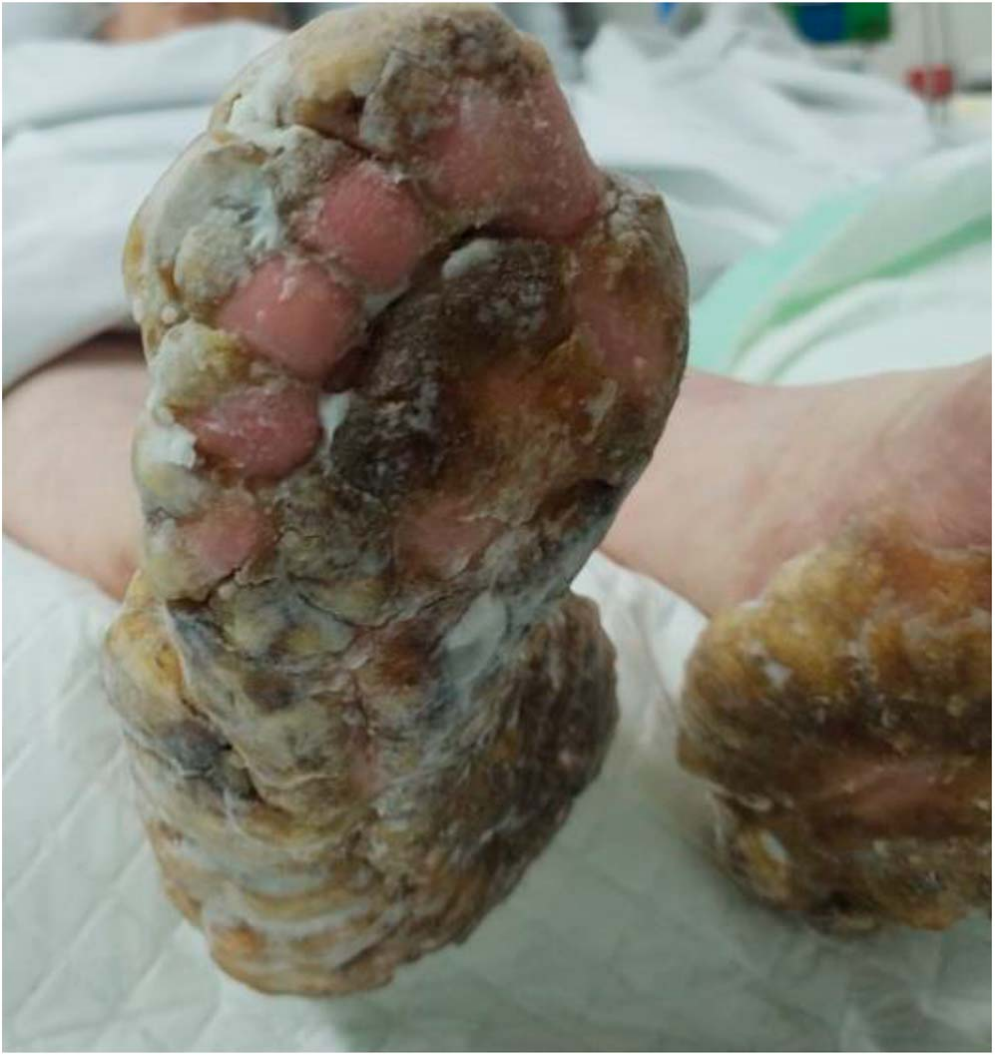
Right foot 2 weeks after using urea.

**Figure 3:**
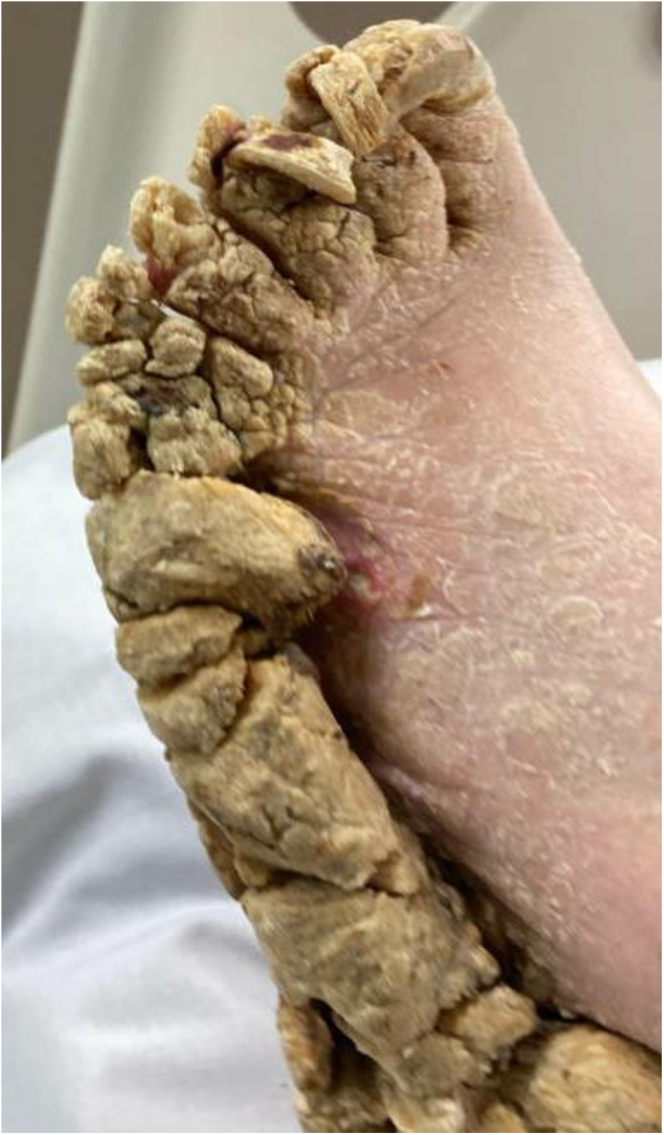
Left foot 2 weeks after using urea.

**Figure 4:**
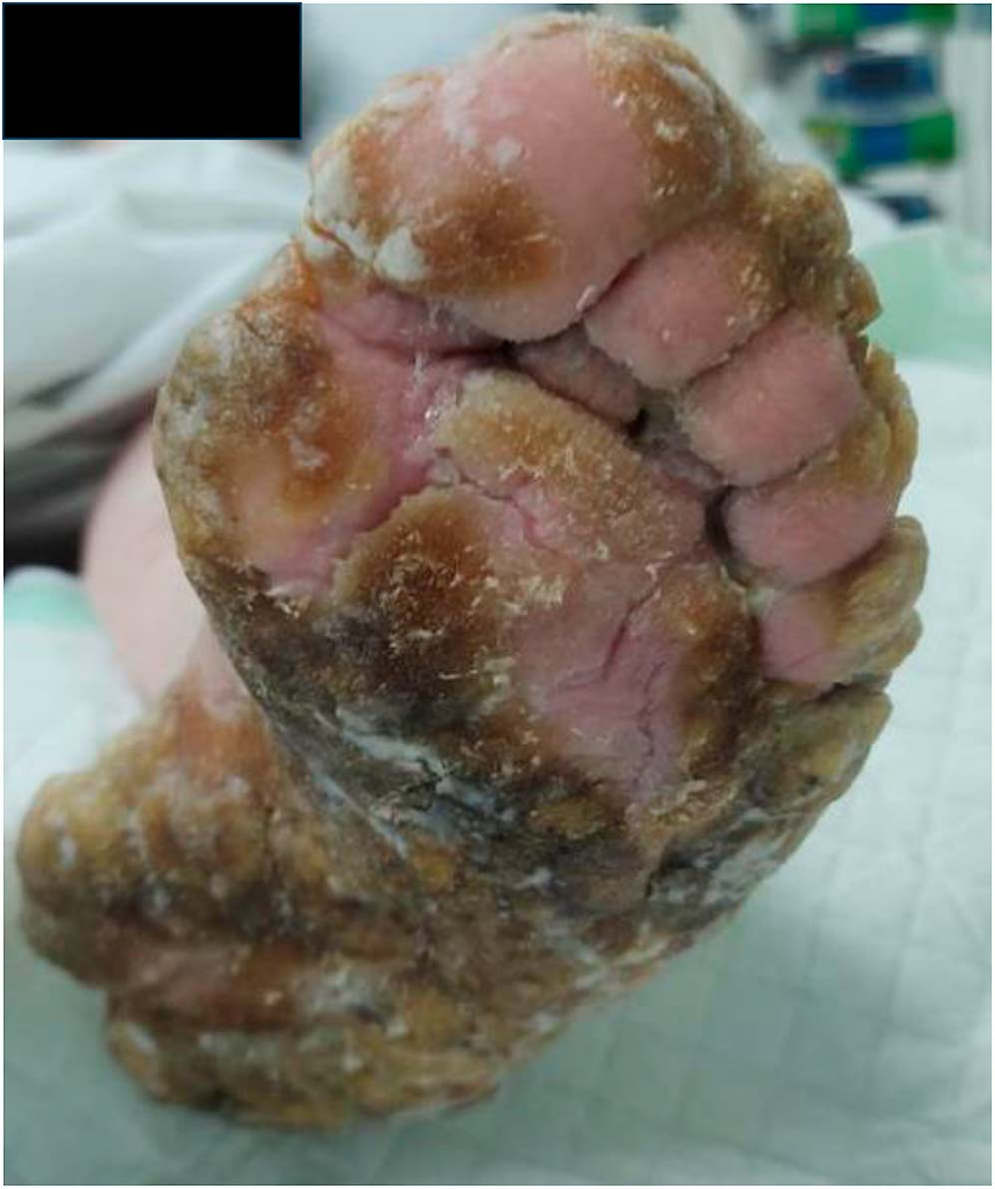
Left foot 2 weeks after using urea.

**Figure 5:**
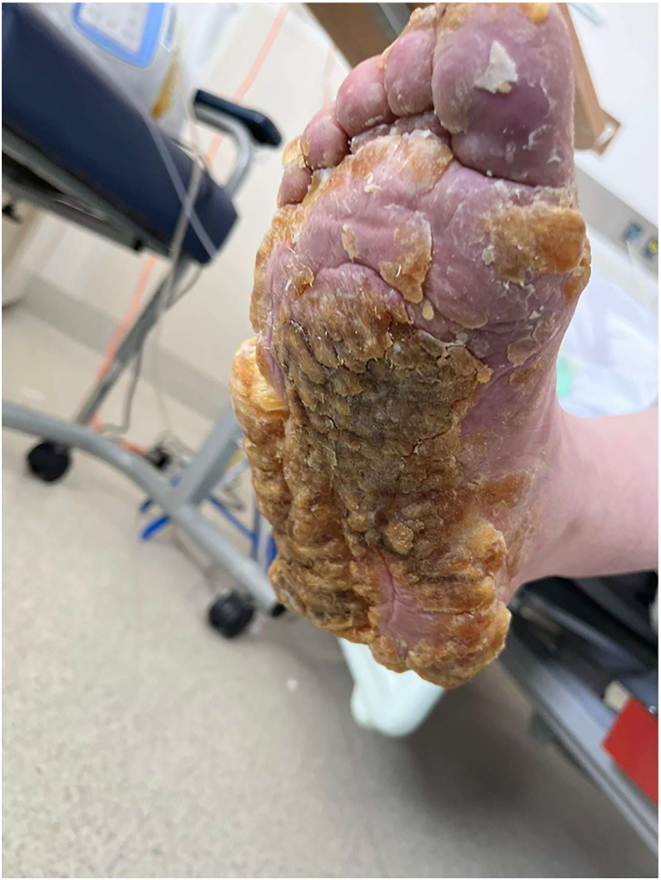
Bottom right foot 1 month after using urea.

**Figure 6:**
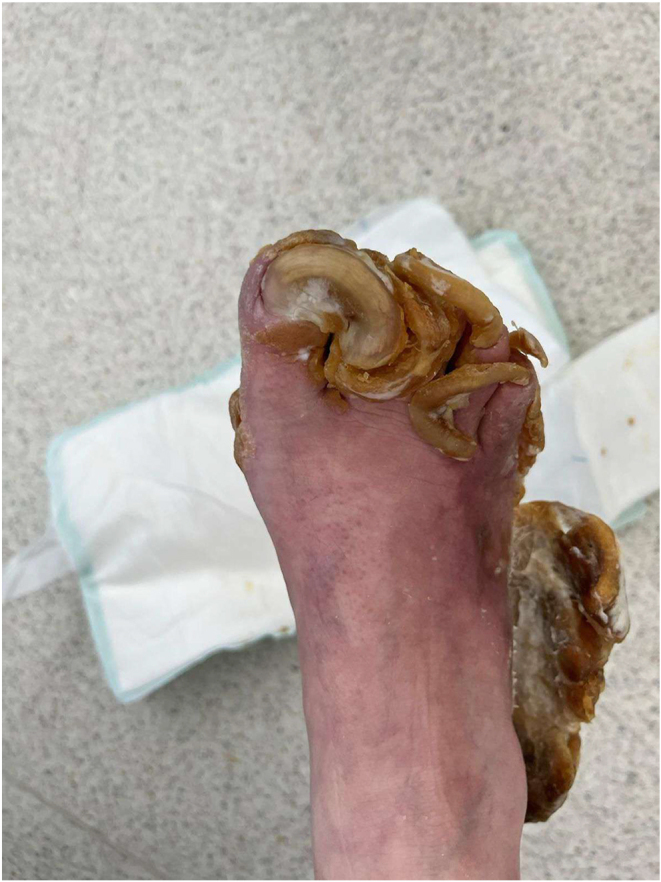
Right foot 1 month after using urea.

**Figure 7:**
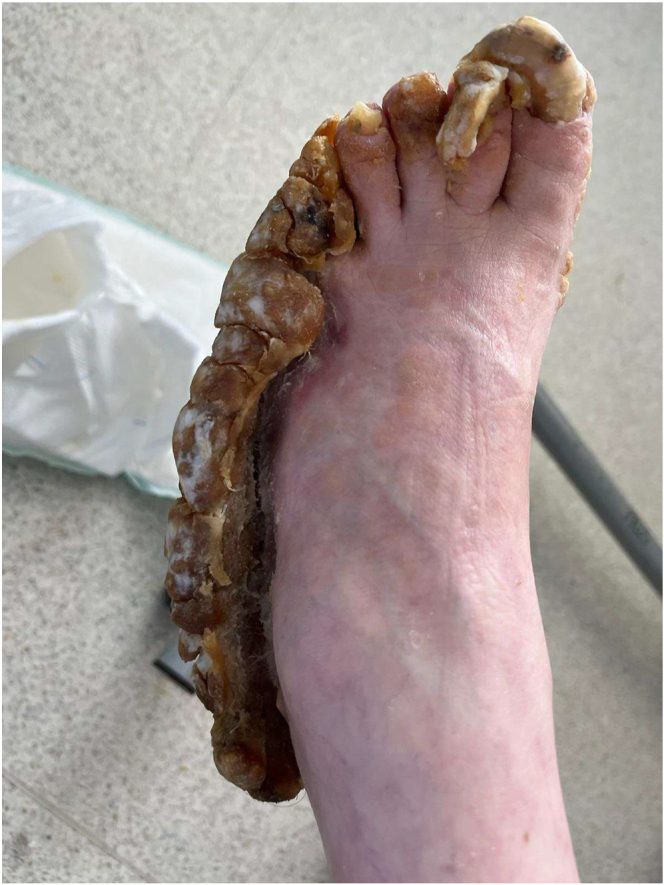
Left foot 1 month after using urea.

**Figure 8:**
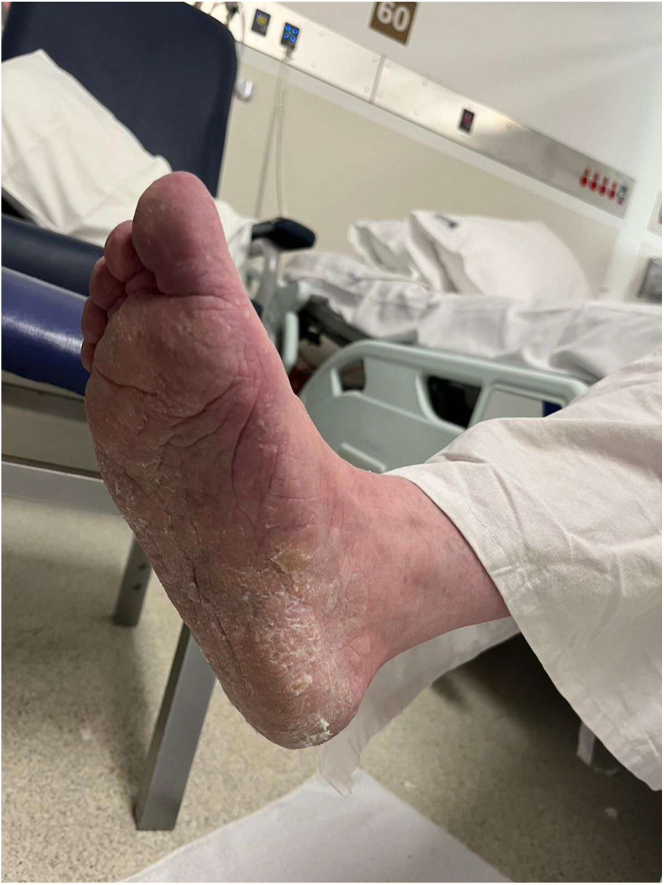
Right foot 2 months after using urea.

**Figure 9:**
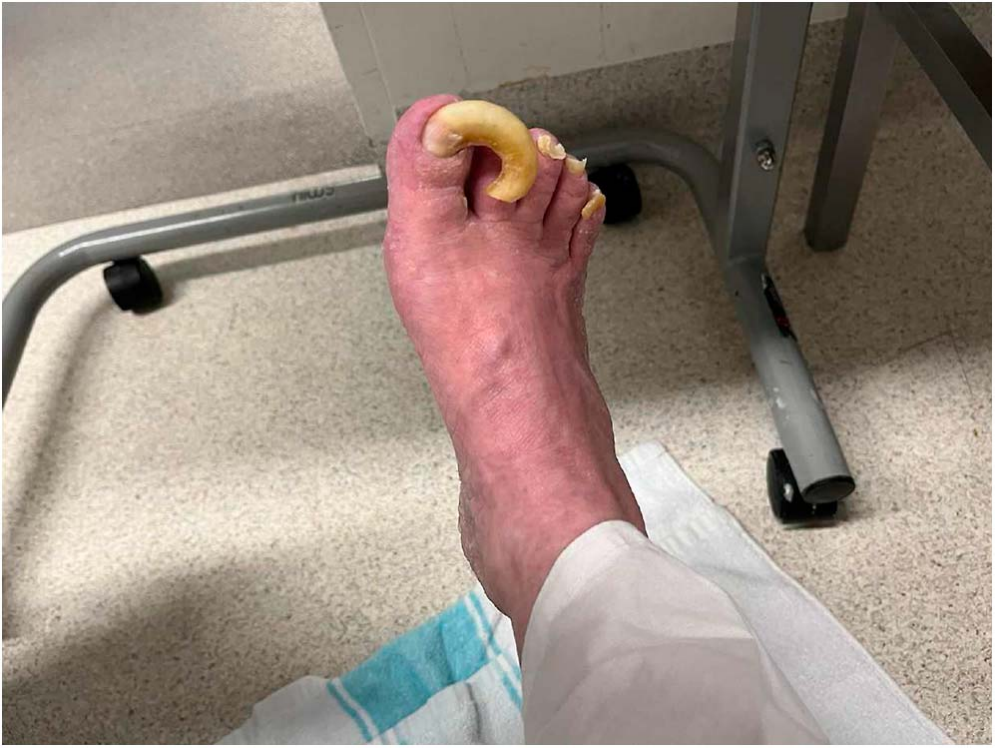
Top right foot 2 months after using urea.

**Figure 10:**
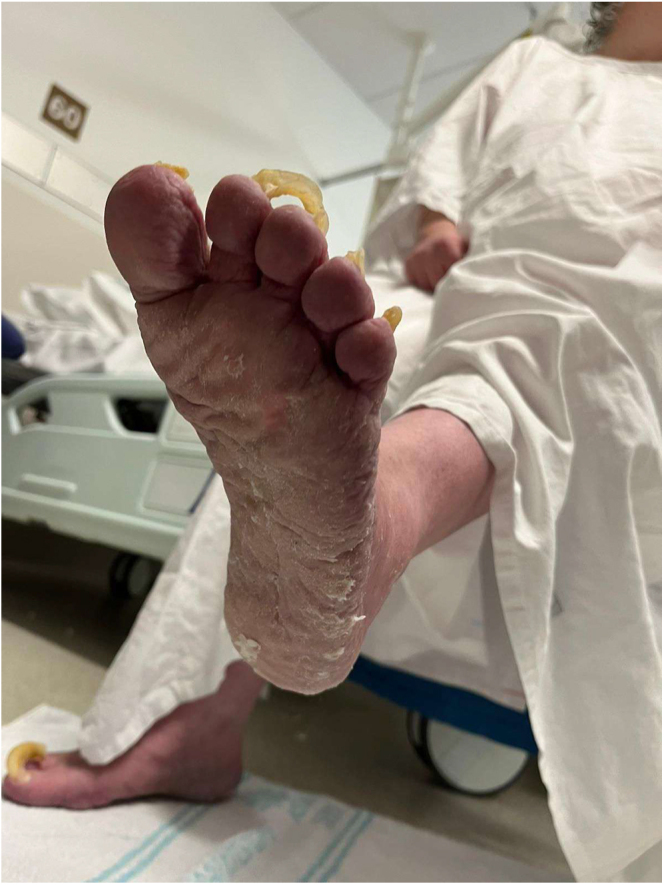
Left foot 2 months after using urea.

The urea cream helped soften the thickened skin, which gradually sloughed off over a few weeks (Figs 2, 3, 4, 5, 6, 7, 8, 9, 10). She was also seen by a podiatrist, who trimmed her toenails. This improved her mobility and enabled her to use a four-wheel walker. She was scheduled for discharge with a plan for early follow-up at the rapid-access diabetes clinic with the multidisciplinary team, followed by the ongoing care at the hospital’s diabetes clinic.

## Discussion

PPK can be either inherited or acquired, presenting in various forms that may also be associated with syndromic conditions. The most common inherited form is diffuse epidermolytic PPK, known as Vorner’s type, which results from an autosomal dominant heterozygous mutation in one of the keratin genes, KRT1 or KRT9. Acquired forms can develop due to skin conditions such as contact dermatitis, psoriasis, eczema and pityriasis rubra pilaris; infections such as tinea pedis; medications such as tyrosine kinase inhibitors; or systemic conditions including bullous pemphigoid, lymphoedema and diabetes (2). Acquired PPK may also appear as a paraneoplastic sign in cancers of the lung, breast, bladder, colon or oesophagus.

The exact mechanism of PPK remains unclear. In this case, no other acquired causes were identified, and the patient declined genetic testing.

Some studies suggest that peripheral neuropathy and repetitive trauma might contribute to the development of keratoderma. In addition, ichthyosiform changes and hyperkeratosis in patients with diabetes could result from advanced glycosylation, which alters skin protein structure. Another hypothesis is that reduced synthesis of structural proteins in insulin deficiency can lead to ichthyosiform changes and keratoderma, a process more common in type 1 diabetes but also possible in type 2 (3). Administering insulin may improve hyperkeratotic skin changes by enhancing the synthesis of these structural proteins.

Treatment primarily aims to soften the skin, thereby reducing symptom burden. Frequent use of emollients is recommended, and patients should be screened for any secondary bacterial or fungal infections, which should be treated appropriately if detected. Pharmacological options include keratolytics, such as topical urea, salicylic acid or lactic acid, and topical or systemic retinoids (2, 4). Some cases of PPK may benefit from weekly soaks, followed by gentle removal using tools such as pumice stones, but this should only be done after consulting a dermatologist. Ongoing podiatry input and follow-up are also recommended.

In patients with PPK, finger prick blood testing can pose a physical challenge, so alternative methods, such as continuous glucose monitoring, should be considered, although they may have financial implications (5).

In this case, acquired PPK was considered to be likely secondary to diabetes due to the absence of other potential causes and lack of consent for genetic screening.

Optimal diabetes control is essential to prevent complications, both macrovascular and microvascular, and cutaneous manifestations, including rarer ones like PPK. Regular follow-up with an endocrinologist, optometrist, diabetes nurse educator and allied health professionals, such as podiatrists, is strongly recommended.

## Declaration of interest

The authors declare that there is no conflict of interest that could be perceived as prejudicing the impartiality of the work.

## Funding

This work did not receive any specific grant from any funding agency in the public, commercial or not-for-profit sector.

## Author contribution statement

The patient was admitted under the care of W N Cheung. F Iqbal helped in the management of the patient. K Phan was on the consulting team providing advice to the patient’s care. All authors were involved in manuscript writing and approved the final draft.

## Patient consent

Written informed consent was obtained from the patient for publication of this case report.
